# Does the application of diffusion weighted imaging improve the prediction of survival in patients with resected brain metastases? A retrospective multicenter study

**DOI:** 10.1186/s40644-020-0295-4

**Published:** 2020-02-07

**Authors:** Rasheed Zakaria, Yin Jie Chen, David M. Hughes, Sumei Wang, Sanjeev Chawla, Harish Poptani, Anna S. Berghoff, Matthias Preusser, Michael D. Jenkinson, Suyash Mohan

**Affiliations:** 1 Department of Neurosurgery, The Walton Centre NHS Foundation Trust, Liverpool, UK; 2 Institute of Integrative Biology, University of Liverpool, Liverpool, UK; 3 Division of Neuroradiology, Department of Radiology, Perelman School of Medicine at the University of Pennsylvania, Philadelphia, USA; 4 Biostatistics, University of Liverpool, Liverpool, UK; 5 Institute of Translational Medicine, University of Liverpool, Liverpool, UK; 6 Department of Medicine I, Division of Oncology, Medical University of Vienna, Vienna, Austria

**Keywords:** Brain metastasis, Cerebral metastasis, Diffusion MRI, DWI, Biomarkers, Survival modelling, Personalised medicine

## Abstract

**Background:**

Brain metastases are common in clinical practice. Many clinical scales exist for predicting survival and hence deciding on best treatment but none are individualised and none use quantitative imaging parameters. A multicenter study was carried out to evaluate the prognostic utility of a simple diffusion weighted MRI parameter, tumor apparent diffusion coefficient (ADC).

**Methods:**

A retrospective analysis of imaging and clinical data was performed on a cohort of 223 adult patients over a ten-year period 2002–2012 pooled from three institutions. All patients underwent surgical resection with histologically confirmed brain metastases and received adjuvant whole brain radiotherapy and/or chemotherapy. Survival was modelled using standard clinical variables and statistically compared with and without the addition of tumor ADC.

**Results:**

The median overall survival was 9.6 months (95% CI 7.5–11.7) for this cohort. Greater age (*p* = 0.002), worse performance status (*p* < 0.0001) and uncontrolled extracranial disease (*p* < 0.0001) were all significantly associated with shorter survival in univariate analysis. Adjuvant whole brain radiotherapy (*p* = 0.007) and higher tumor ADC (*p* < 0.001) were associated with prolonged survival. Combining values of tumor ADC with conventional clinical scoring systems such as the Graded Prognostic Assessment (GPA) score significantly improved the modelling of survival (e.g. concordance increased from 0.5956 to 0.6277 with Akaike’s Information Criterion reduced from 1335 to 1324).

**Conclusions:**

Combining advanced MRI readings such as tumor ADC with clinical scoring systems is a potentially simple method for improving and individualising the estimation of survival in patients having surgery for brain metastases.

## Background

Brain metastases (BM) are an increasing clinical challenge causing significant morbidity and mortality [1]. Multiple treatments are available including neurosurgical resection, radiotherapy, radiosurgery, chemotherapy and immunotherapy. The variety of available treatments can make it difficult to formulate a patient-specific treatment plan. To help, simple prognostic models based on clinical information were developed, including the Recursive Partitioning Analysis (RPA) scale [2] and Graded Prognostic Assessment (GPA) score [3]. Recent schemes have added primary cancer (the disease-specific GPA [4]) and biological information such as receptor pathway status and biochemical parameters [5–8] but no study has added advanced quantitative imaging measures to try and improve these models.

Although commonly used, the performance status - which is the major factor in these scores - is subjective and can vary between visits due to confounders such as corticosteroid use. There is a need for objective, non-invasive biomarkers that reflect the intrinsic biologic behaviour of the tumor. Diffusion weighted imaging (DWI) is a rapidly obtained sequence in clinical practice and is an accepted part of standard brain tumor imaging [9]. Single center studies have identified apparent diffusion coefficient (ADC) values within the BM as a particularly promising marker; tumor ADC correlated with survival and recurrence after surgical resection [10] and survival after radiosurgery [11], whilst ADC changes at the tumor edge may indicate a more locally aggressive phenotype [12].

Therefore, it is logical to add tumor ADC to traditional clinical scores like RPA, GPA to determine whether this improves the prediction of survival. This would be of immense clinical value in helping to select the appropriate treatments for the individual patient based on their prognosis – personalised medicine – without the need for further invasive tests or procedures. Also as a proof of concept for applying an advanced MRI biomarker in this additive way, we are perhaps opening up the possibility of including it in future larger studies.

This study retrospectively pooled clinical and radiological data of patients with BM from three institutions and compared survival models using just the standard clinical factors with those using standard clinical factors *plus* an imaging biomarker, the tumor ADC. This allowed an evaluation of whether the imaging marker could *improve* the prognostication of overall survival in patients with surgically resected BM. In addition to providing sufficient numbers for statistical analysis, the collaborative nature of the project across three different institutions will help in establishing the external validity of the results, assess any differences in acquiring the imaging marker between large centers and identify obstacles that may hinder its implementation in clinical practice.

## Methods

### Study design, setting and participants

This was a retrospective study of 223 adult patients with resected BM from solid organ cancers across three specialist departments in Austria (Medical University of Vienna, VIE, *n* = 30), the United States of America (University of Pennsylvania, PENN *n* = 100) and the United Kingdom (Walton Centre Liverpool, LIV, *n* = 93) over a 10 year period 2002–2012. Patient demographics and relevant clinical details about the studied population are listed in Table 1. A retrospective study design was chosen as it meant that patients with complete survival data and MRI data were immediately available to test the hypothesis. All cases in this time period who had undergone surgical resection of a solitary BM (multiple metastases were included only when there was a single dominant metastasis being operated upon along with other smaller lesions, reflecting the real life situation) were included. Exclusions were any patient without a preoperative DWI study or patients who had previous radiation therapy either local or whole brain as this could have altered the diffusion characteristics. Limiting the study to surgically resected metastases meant that the diagnosis was certain (with pathology being the gold standard), there was no variation in treatment (standard of care was surgery then whole brain radiotherapy (WBRT) and this study period predates the use of cavity radiosurgery, immunotherapy, laser interstitial therapy). The most widely used scores for predicting survival in tumor boards, multidisciplinary team meetings and clinics were calculated using the clinical information - the GPA, disease-specific GPA and RPA score [2–4].

**Table 1:** Demographic and clinical summary (*n* = 223)

Factor	Median	IQ range
Age	59 years	52–67
	Category	Count (% of total)
Gender	Male	106 (48%)
	Female	117 (52%)
KPS	> 70	162 (73%)
	< 70	61 (27%)
Primary cancer	Lung	115 (52%)
	Breast	34 (15%)
	Melanoma	18 (8%)
	Kidney	7 (3%)
	Colorectal	10 (4%)
	Unknown primary	2 (1%)
	Other	37 (17%)
Status extracranial disease	Complete response	36 (16%)
	Partial response	4 (2%)
	Stable disease	26 (12%)
	Progressive disease	28 (13%)
	No evidence of disease	41 (18%)
	Synchronous	87 (39%)
Number of brain metastases	1	158 (71%)
	2	30 (13%)
	3	11 (5%)
	> 3	24 (11%)
RPA Class	I	62 (28%)
	II	151 (68%)
	III	10 (4%)
GPA score	0-1	26 (12%)
	1.5 - 2	108 (48%)
	2.5 - 3	53 (24%)
	3.5 - 4	36 (16%)
DS-GPA score	0-1	29 (16%)
	1.5 - 2	111 (60%)
	2.5 - 3	36 (19%)
	3.5 - 4	10 (5%)
WBRT after neurosurgery (complete resection)	No	76 (34%)
	Yes	144 (65%)
	Unknown	3 (1%)

*KPS* Karnofsky performance status, *RPA* recursive partitioning analysis, *GPA* graded prognostic assessment, *DS-GPA* disease specific GPA, *WBRT* whole brain radiotherapy

### Imaging acquisition and analysis

**Fig. 1:**
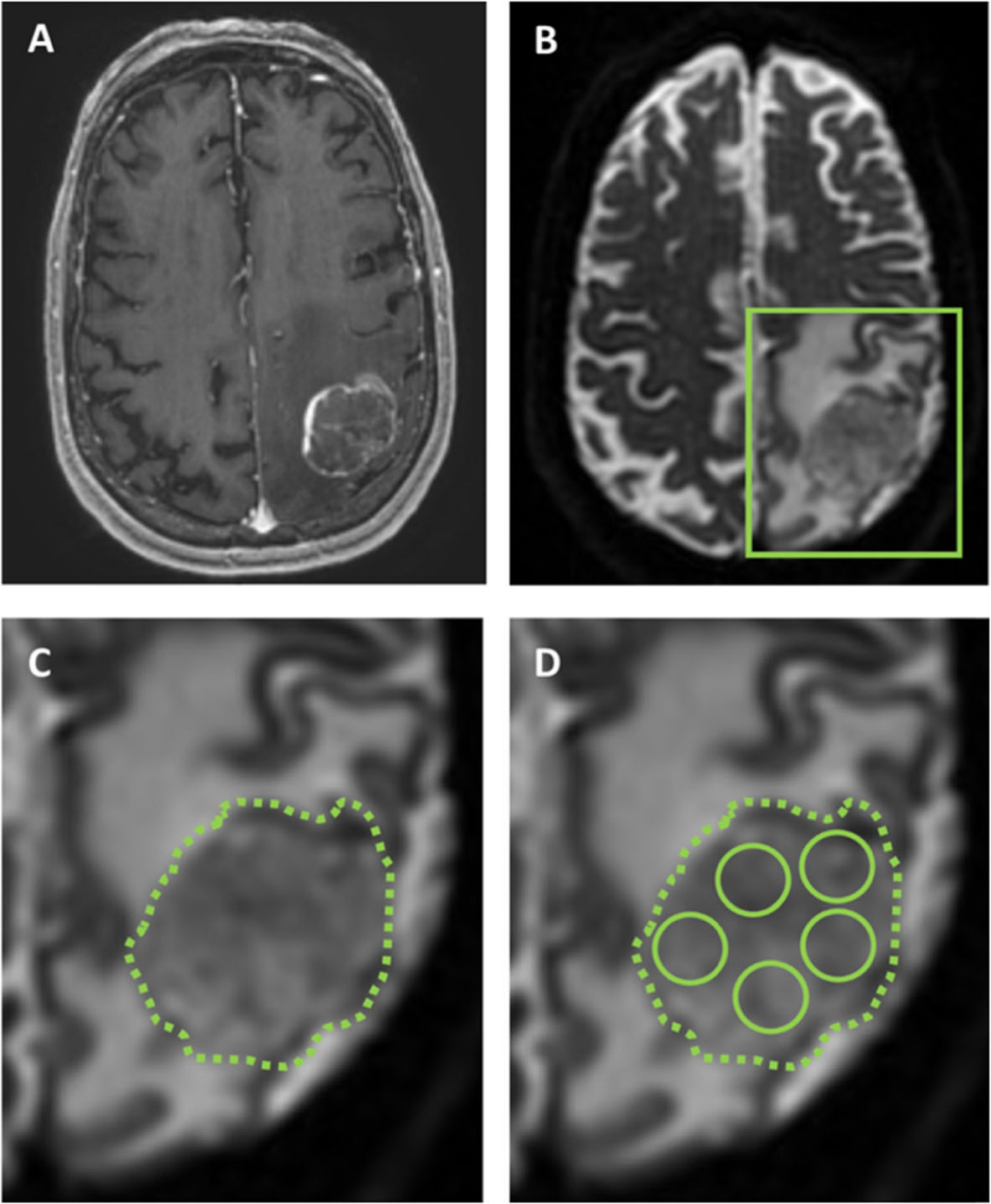
Example of measurement of tumor ADC by manual placement of regions of interest A patient with a history of lung adenocarcinoma presents with headache and focal neurological deficit. **a**. T1 weighted MRI with gadolinium demonstrates a left parietal lesion, which was confirmed as a metastasis by pathology. **b**. ADC map is generated from DWI using post processing software and fused to the T1 post contrast study. C and D are zoomed images of B as outlined in green. **c**. A region of interest is traced around the tumor border using the T1-weighted post gadolinium scan and then applied to the ADC map. **d**. Regions of interest of an agreed size - here five circles of 50mm^2^ -are placed within the tumor on the axial slice with the largest area, avoiding necrosis, haemorrhage or cyst, and the overall mean is calculated

As this was a pragmatic retrospective study, post-contrast T1 and DWI sequences had already been obtained preoperatively *as per each institution’s local protocol*. For PENN, DWI was acquired at 3Tesla using a single shot spin-echo echo planar; TR/TE 5000/86 ms, FOV 22 × 22 cm, 3 mm slice thickness, 128 × 128 matrix, 30 diffusion directions. For LIV: two sequences were used at 3Tesla, a spin echo DwiSE sequence, TR/TE 2828/73 ms, FOV 23x23cm, 4 mm slice thickness, 128 × 128 matrix and 32 diffusion directions or DTI with TR/TE 8000/87.8 ms, FOV 24x24cm, 4 mm slice thickness, 128 × 128 matrix and 25 directions. For VIE: DWI examinations were performed at 1.5Tesla and 3Tesla. Imaging parameters varied due to different scanners; slice thickness, range 3 - 6 mm; FOV, range 128 × 128 – 384 × 384. Diffusion weighting was applied with ‘b’ values of 0 and 1000 s/mm^2^ in all institutions. Image processing has been described in previous studies and used standard techniques, relying on the contrast-enhanced T1-weighted scan for delineating the margins of the BM. Mean tumor ADC was calculated from the contrast enhancing tumor avoiding cystic or haemorrhagic areas. An example of one technique using circular regions of interest (ROI) (each 50 mm^2^) is illustrated in Fig. 1. ADC readings were taken by a single radiology trained researcher at each institution blind to the clinical outcomes although we have previously demonstrated there is good intra and inter-rater reliability for this type of measurement amongst radiologists and trained researchers [13]. Only the largest, resected BM was assessed for those with multiple lesions (note that in general the cases with more than one metastasis included a dominant lesion with only small additional lesions).

### Statistical methods

MRI data were processed at each individual institution, then MRI results along with clinical data were anonymized and statistical analysis performed at one institution by one statistics researcher with expertise in survival analysis and modelling (DH at LIV). Overall survival (OS) from initial diagnosis of BM to death was calculated, censored at the last recorded clinical contact. In univariate analysis differences in OS were examined, using log rank tests, based on each of the factors listed in Table 1. Data were stratified by center to account for potential confounding differences in treatment, MRI acquisition parameters and follow up between institutions. A Cox proportional hazards model was fitted for each of GPA, RPA, WBRT and ADC separately and then all combinations of these variables. The goal was to examine which model best described overall survival. In multivariate analysis, the best fitting Cox proportional hazards models were selected using variable reduction by Akaike’s Information Criterion (AIC) [14], which is a way of measuring concordance (how well the model fits the data) whilst penalising for extra parameters. Specifically, a low AIC value indicates a more accurate model. Statistical analysis was performed using R version 3.21 (R Core Team, 2013).

## Results

### Clinical outcomes for this population

Median overall survival was 9.6 months (95% CI 7.7–11.7). A total of 187 deaths were observed in our cohort (83.9%) during a median follow up of 9.6 months (interquartile range = 4.5 to 17.3 months, minimum follow up = 7 days, maximum follow up = 8 years). Greater age (hazard ratio [HR] for death = 1.02, 95% CI 1.01–1.04, *p* = 0.002), worse KPS (HR = 1.03, 95% CI 1.02–1.05, *p* < 0.0001) and uncontrolled extracranial disease (partial, progressive or synchronous vs. complete response, HR = 2.44, 95% CI 1.47–4.06, *p* < 0.0001) were all significantly associated with shorter survival in univariate analysis stratified by institution. A likelihood ratio test from a univariate cox model (stratified by institution) with number of BM as the independent predictor did not show a significant relationship between number of metastases and time to death (*p* = 0.156). A likelihood ratio test from a univariate cox model (stratified by institution) with cancer type as the independent predictor did not show a significant relationship between cancer type and time to death (*p* = 0.0573). GPA (I vs. II *p* = 0.02, I vs. III *p* = 0.001, I vs. IV 0.154) and RPA (class I vs. II, *p* = 0.0016, I vs. III *p* = 0.02) categories were significantly associated with differences in survival in univariate analysis. Disease specific-GPA data was not significant on non-stratified analysis (*p* = 0.114), therefore it was not considered further. Adjuvant WBRT (bearing in mind this was a historical series) was administered in 144/223 of patients (64.6%) and was associated with significantly longer survival (*p* = 0.007) when stratified by center; therefore, it was included in the subsequent multivariate analyses.

**Fig. 2:**
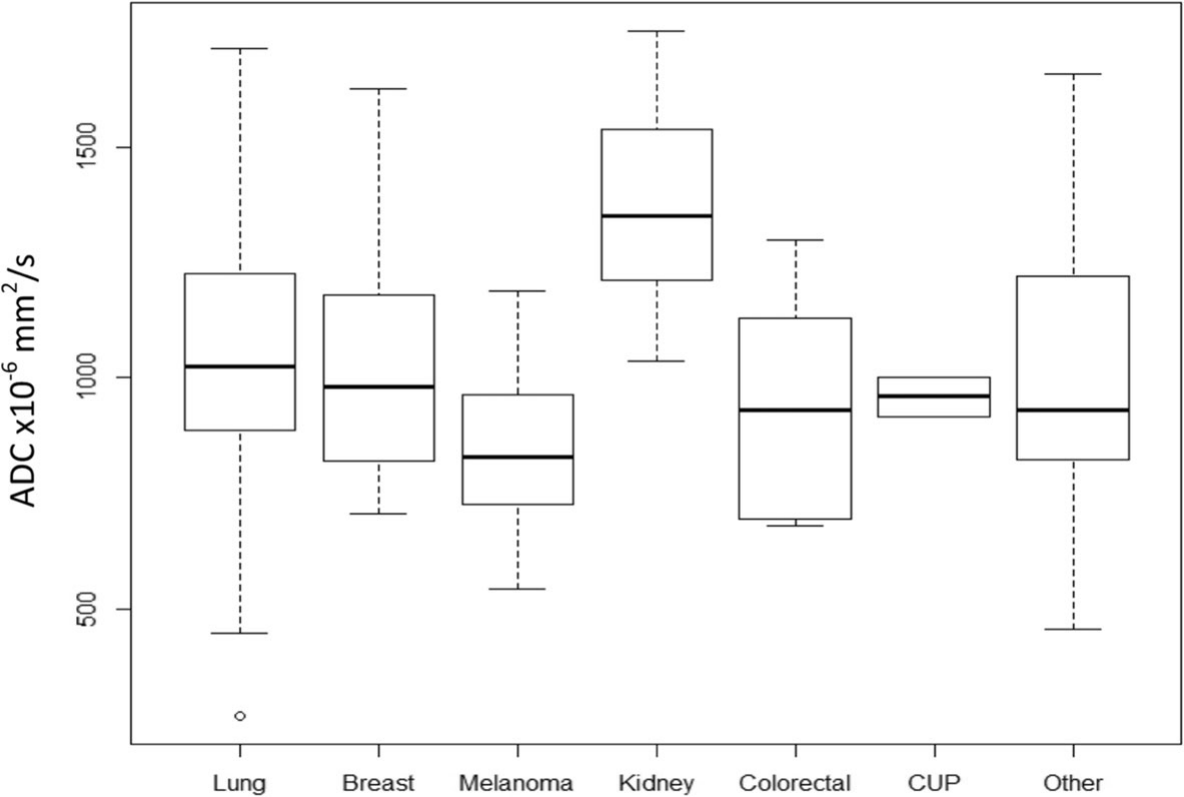
Tumor ADC of 223 brain metastases by primary cancer type. The ADC values varied with cancer type (ANOVA ADC x primary cancer, *p* = 0.001) but it was not possible to distinguish the most common brain-tropic cancers: lung, breast and melanoma (Tukey HSD statistic breast vs. lung = − 9.7, breast vs. melanoma = − 184.5, lung vs. melanoma = 194.2, *p* > 0.05 all comparisons). CUP = cancer of unknown primary. Only 7 renal cell carcinoma cases were included so the higher tumor ADC in this group leading to the overall ANOVA result may be a sampling effect. Primary cancer itself was not associated with overall survival in this series (see Results)

*Imaging biomarker and influence on clinical models:* The median tumor ADC value was 1.001 × 10^− 3^ mm^2^/s (range 0.268–1.753). The mean tumor ADC was 1.025 × 10^− 3^ mm^2^/s, standard deviation 0.269. The ADC values varied with cancer type (ANOVA ADC x primary cancer, *p* = 0.001). As seen in Fig. 2, with regards to the common clinical question of “what is the likely primary?”, it was not possible to distinguish the most common brain-tropic cancers using tumor ADC: lung, breast and melanoma (Tukey HSD statistic breast vs. lung = − 9.7, breast vs. melanoma = − 184.5, lung vs. melanoma = 194.2, *p* > 0.05 all comparisons). Tumor ADC was found to be significantly correlated with survival when treated as a continuous covariate (HR = 0.99, for a unit change of 1 in ADC), with a higher tumor ADC correlating with longer survival (*p* < 0.001). Also, each time ADC was added to a model, it decreased AIC suggesting that it improves the model fit. Each time ADC was added to a model, the predictive ability improved as shown by the improved concordance (Table 2**)**. To further investigate whether this improvement in the model was actually due to the addition of ADC (and that ADC was not just a surrogate for cancer type), data was re-analysed for only lung cancer patients, given this was the most common tumor type in our series (*n* = 115). For the lung cancer cohort, the mean tumor ADC is 1.039 × 10^− 3^ mm^2^/s (SD 0.262) and the median is 1.025 × 10^− 3^ mm^2^/s (range 0.268–1.713). The same results were observed, with improved fit each time ADC was added (Table 3**)**.

**Table 2:** Comparison of models for predicting overall survival in brain metastases

Model	AIC	Concordance	R^2^
Graded Prognostic Assessment (GPA)	1335.30	0.5956	0.1058
Recursive Partitioning Analysis (RPA)	1328.94	0.5999	0.1232
GPA + Tumor ADC	1324.48	0.6277	0.1558
RPA + Tumor ADC	1321.84	0.6240	0.1582
GPA + WBRT + Tumor ADC	1292.37	0.6545	0.1834
RPA + WBRT + Tumor ADC	1290.61	0.6662	0.1825

*WBRT* received adjuvant whole brain radiotherapy

**Table 3:** Survival modelling for lung cancer cases alone (*n* = 115)

Model	AIC	Concordance	R^2^
Graded Prognostic Assessment (GPA)	594.92	0.5477	0.0225
Recursive Partitioning Analysis (RPA)	581.92	0.5719	0.1116
Tumor ADC	581.94	0.5771	0.0959
GPA + Tumor ADC	584.93	0.5862	0.1192
RPA + Tumor ADC	575.08	0.5974	0.1773
GPA + WBRT + Tumor ADC	578.52	0.6363	0.1813
RPA + WBRT + Tumor ADC	569.58	0.6523	0.2293

*WBRT* received adjuvant whole brain radiotherapy

## Discussion

### Summary

This multicentre retrospective study suggests that for a specific population of surgically resected brain metastases, an advanced quantitative MRI based biomarker – the tumor ADC - improves the prediction of overall survival when added to the standard, widely used clinical indexes such as recursive partitioning analysis (RPA) and graded prognostic assessment (GPA) score.

### What new information this adds to the field

In this study, we have demonstrated that even in a multi-institutional series using standard of care clinical MRI techniques, there is a significant association of tumor ADC with length of survival after resection and improvement of existing clinical scores for predicting survival by incorporating tumor ADC values. Predicting prognosis is critical in patients with BM, not only to guide management discussions between clinicians and patients, but also to stratify patients for randomised trials. The ADC values from three international institutions were similar in range and variance to the values reported in the literature [12, 15, 16]. This cohort is representative of routine clinical practice with respect to demographics, proportion of primary cancer types and overall survival [1, 17]. There was also a clear separation of patient survival based on the standard RPA and GPA indices, lending further validity to our patient cohort. Finally the effect we observed persisted even when just lung cancer cases were analysed, suggesting that the tumor ADC is not simply a surrogate of primary.

### Limitations

From a technical point of view, we selected tumor ADC, since single center studies of BMs have previously shown an association of higher tumor ADC with improved survival or delayed recurrence after surgery or radiosurgery [10, 12, 15, 16, 18], and this measurement is simple to obtain in clinical practice. It would have been better if the MRI data could have been uploaded centrally and then analysis of ADC maps performed centrally, perhaps by two researchers with some reliability analysis. However, this is not what would happen in clinical practice if this measurement were adopted and placing ROIs over the tumor on clinical workstations is something radiologists could reasonably do in the existing clinical workflow without the need of specialised software. To maximize the generalizability of our study results, real, retrospective data obtained at different institutions in different countries were utilized and as a result details about tumor volume, location and size were not available to be analysed as confounders. In general, as these were resected tumors they were all likely to be large or else they would have had SRS and in accessible rather than deep locations (again, that would likely have favoured SRS). Tumor volume may affect ADC and this is why the method explicitly uses multiple areas of reading and avoids necrosis, which is likely to be more common and centrally located in larger tumors. For the same reasons, retrospective information about adjuvant chemotherapy regimes were not available universally, although whole brain radiation was documented and analysed. Chemotherapy in general is poorly effective against BM although it may have affected overall survival which reflects systemic disease. Extracranial disease control was able to be analysed as it is a potential important confounder, and since this was a historical series, transformative treatments like immunotherapy which would certainly have had a huge impact on overall survival - e.g. for melanoma cases - were not in use. Primary cancer type did not seem to influence survival in this cohort therefore differences in treatment of the different primary cancers (e.g. radioresistant vs. radiosensitive types or targeted agents) did not seem be a confounder.

### Biological significance and future directions

Although in stroke cases ADC has been shown to vary with MRI coils, vendors and field strength [19] DWI data appears to be comparable across vendors and institutions [20, 21]. Practical measures such as using multiple readers, standard derivations of measures and even cloud based post-processing platforms for central processing of raw data are all likely to minimise variation in future studies. Nonetheless, even the current, widely used GPA and RPA scales can suffer from inter-observer variability, such as the subjective nature in which KPS is determined. Tumor ADC is widely studied in other solid organ cancers as a biomarker of survival [22] although paired values for primary and BM have not been reported, it would be interesting to determine which influence overall survival more. High tumor ADC has been correlated with lower tumor cellularity [16], reduced extracellular matrix density [10] and greater degree of tumor differentiation [12] in BM and any of these could reasonably be surrogates of improved survival. We do not suggest that quantitative imaging biomarkers alone will replace the traditional clinical factors that have demonstrated utility across large numbers of patients over many years and changes in treatment modalities. Rather, we propose that quantitative imaging biomarkers be added to these clinical indices towards improving and personalising prognostication. Finally, the treatment paradigms in BM are currently undergoing great change with neo-adjuvant and adjuvant cavity SRS, immunotherapy and prophylactic chemotherapy for asymptomatic micrometasteses all being reported. This may make clinical models predicting prognosis obsolete. The solutions will be to re- apply these models to each new cohort (e.g. does pre-op tumor ADC improve survival modelling for surgery + cavity SRS patients too?), which is exactly how they were refined and developed originally *and* to embrace the opportunity to incorporate these individualised, non-invasive biomarkers provided by advanced MRI techniques (e.g. by testing MRI features as a measure of immunotherapy response as has been tried recently with post contrast features in a radiomics approach [23]).

## Conclusions

We have demonstrated that diagnostic MRI scans including DWI sequences contain significant biological information which can be incorporated with standard clinical parameters to improve the prediction of overall survival in patients with surgically resected BM and better inform clinical decision making.

## Data Availability

No additional data.

## Abbreviations

ADC: Apparent diffusion coefficient
AIC: Akaike’s Information Criterion
BM: Brain metastases
DWI: Diffusion weighted MRI
GPA: Graded prognostic assessment
OS: Overall survival
RPA: Recursive partitioning analysis
WBRT: Whole brain radiotherapy
